# Effects of environmental enrichment on white matter glial responses in a mouse model of chronic cerebral hypoperfusion

**DOI:** 10.1186/s12974-017-0850-5

**Published:** 2017-04-11

**Authors:** Yoshiki Hase, Lucinda Craggs, Mai Hase, William Stevenson, Janet Slade, Dianne Lopez, Rubin Mehta, Aiqing Chen, Di Liang, Arthur Oakley, Masafumi Ihara, Karen Horsburgh, Raj N. Kalaria

**Affiliations:** 1 Neurovascular Research Group, Institute of Neuroscience, Newcastle University, Campus for Ageing and Vitality, Newcastle upon Tyne, NE4 5PL UK; 2 Department of Stroke and Cerebrovascular Diseases, National Cerebral and Cardiovascular Centre, Osaka, Japan; 3 Centre for Neuroregeneration, University of Edinburgh, Edinburgh, UK

**Keywords:** Animal model, Chronic cerebral hypoperfusion, Clasmatodendrosis, Environmental enrichment, Glial activation/proliferation

## Abstract

**Background:**

This study was designed to explore the beneficial effects of environmental enrichment (EE) on white matter glial changes in a mouse model of chronic cerebral hypoperfusion induced by bilateral common carotid artery stenosis (BCAS).

**Methods:**

A total of 74 wild-type male C57BL/6J mice underwent BCAS or sham surgery. One week after surgery, the mice were randomly assigned into three different groups having varied amounts of EE—standard housing with no EE conditions (std), limited exposure with 3 h EE a day (3 h) and full-time exposure to EE (full) for 12 weeks. At 16 weeks after BCAS surgery, behavioural and cognitive function were assessed prior to euthanasia. Brain tissues were analysed for the degree of gliosis including morphological changes in astrocytes and microglia.

**Results:**

Chronic cerebral hypoperfusion (or BCAS) increased clasmatodendrocytes (damaged astrocytes) with disruption of aquaporin-4 immunoreactivity and an increased degree of microglial activation/proliferation. BCAS also impaired behavioural and cognitive function. These changes were significantly attenuated, by limited exposure compared to full-time exposure to EE.

**Conclusions:**

Our results suggest that moderate or limited exposure to EE substantially reduced glial damage/activation. Our findings also suggest moderate rather than continuous exposure to EE is beneficial for patients with subcortical ischaemic vascular dementia characterised by white matter disease-related inflammation.

## Background

Dementia resulting from cerebrovascular disorders is the second most prevalent type of dementia [1] which currently has no cure. Therefore, effective and safe therapeutic strategies, which delay or slow the progression of vascular dementia (VaD) symptoms, are urgently required. Subcortical ischaemic vascular dementia (SIVD), the most common subtype of VaD, has a strong relationship to vascular cognitive impairment (VCI) [2]. SIVD is characterised by lacunar infarcts and white matter (WM) lesions [3, 4] and plays a key role for VCI [5]. Cerebral hypoperfusion is defined as decreased cerebral blood flow (CBF) evident in VaD, which may promote WM lesions [2], frontal hypo-metabolism [6] and cognitive impairment [7]. Chronic cerebral hypoperfusion, a major component of SIVD and diffuse WM changes caused by small-vessel disease (SVD), is also strongly correlated with VCI in SIVD patients [8, 9]. However, little is known about the neuropathological mechanisms that lead SIVD patients to progress to VCI.

Animal models of chronic cerebral hypoperfusion have been studied in rats [10–13] and gerbils [14] in order to understand the neuropathological processes that occur in the progression of SVD. Our research group has established several relevant models of VCI/VaD by assessing chronic cerebral hypoperfusion models in rodents [15–20]. Notably, a mouse model of chronic cerebral hypoperfusion induced by common carotid artery stenosis (BCAS) has been evaluated as one of the most reliable rodent models of VaD [21–23]. These models exhibited glial changes, i.e. astrogliosis and microglial proliferation in WM.

Astrocytes are a major factor in the maintenance of brain homeostasis and can be neuroprotective by reducing reactive oxygen species following brain ischaemia [24]. However, once astrocyte morphology is changed in aged humans and rodents [25], proliferated astrocytes, which is indicative of reactive astrogliosis [26], may have detrimental effects in response to various brain injuries, i.e. stroke [27] and enhance neuronal damage [28].

Chronic neurodegenerative diseases are accelerated by inflammatory response, and both microglia and astrocytes are accountable for this inflammatory response in the brain, i.e. glial proliferation and activation [29]. Following neuronal or glial damage, inflammatory response, characterised by microglial activation/proliferation, has been demonstrated as having both beneficial and detrimental effects in various neurological diseases, e.g. stroke and Alzheimer’s disease [30]. However, chronic over-activation of microglia may cause progressive neurotoxic effects in the brain [31].

The environmental enrichment (EE) paradigm has been established to determine environment-induced plasticity through, e.g. social interaction and physical activity [32]. EE has been demonstrated to improve cognitive impairment in humans, and possibly reverse WM damage. There is some evidence that EE could enhance synaptic plasticity and attenuate cognitive deficits in rodents [33–36] and man [37]. These studies reported that EE increased brain plasticity, enhanced neurogenesis, and increased synaptogenesis as well as preserving motor and cognitive function. EE has also been reported to enhance recovery after stroke injury by attenuating microglia-related inflammatory responses via the Toll-like receptor 2 (TLR2) [38]. However, although several previous studies reported the beneficial effects of EE in rodents and man, few studies explored the protective effects of graded degrees of EE against glial damage in WM and possibly VaD.

This study investigated the effects of EE on glial damage and pathological sequelae in a mouse model of chronic cerebral hypoperfusion induced by bilateral common carotid artery stenosis (BCAS), with the aim of identifying effective and safe novel interventional strategies for VCI/VaD. We focused on the question of whether glial responses in the WM induced by BCAS can be modified by readily implemented EE intervention and therefore SIVD-related cellular inflammatory responses.

## Methods

### Animals and surgical procedures

**Fig. 1:**
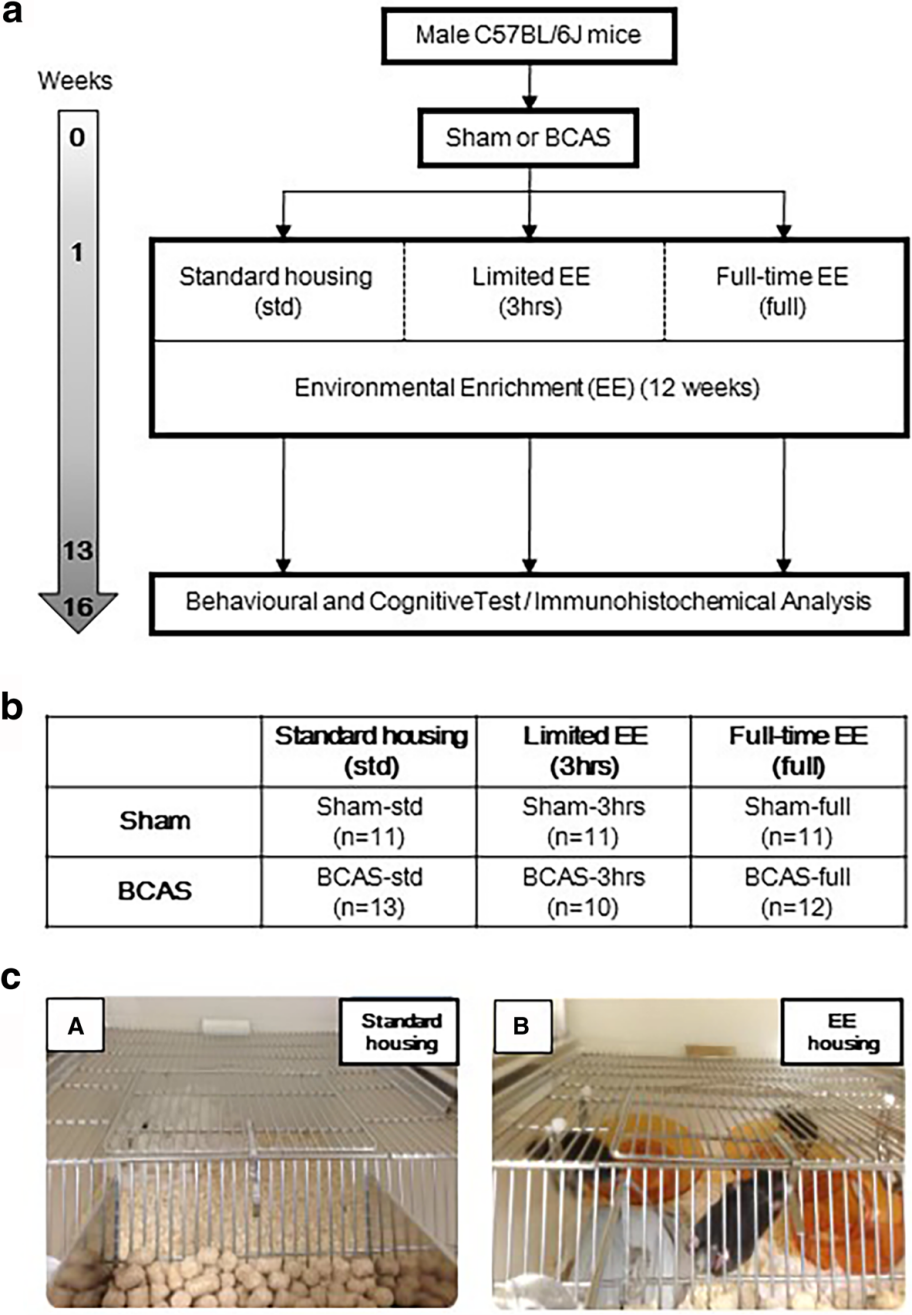
Experimental protocol and animal groups. **a** Experimental protocol − BCAS, bilateral common carotid artery stenosis; EE, environmental enrichment. **b** Animal groups and the number of animals in each group. Sham-std, *n =* 11; Sham-3 h, *n =* 11; Sham-full, *n =* 11; BCAS-std, *n =* 13, BCAS-3 h, *n =* 10; BCAS-full, *n =* 12. **c**(*A* and *B*) Images of standard housing cage (*A*) and EE cage (*B*)

Figure 1a shows the experimental paradigm of the study. Male C57BL/6J mice (9 weeks old, 23.1−25.3 g) were purchased from the Jackson Laboratory, USA. The mice were housed within a 12-h day and 12-h night cycle (6 am–6 pm, day; 6 pm–6 am, night) and were given access to food and water ad libitum. After 1-week acclimatisation, a total of 74 mice were randomly chosen for either bilateral common carotid artery stenosis (BCAS, *n =* 41) or sham (*n =* 33) surgery. BCAS surgery was performed as described previously [15]. Briefly, mice were anaesthetised by 1.5% isoflurane in oxygen and air. A middle neck incision was made; bilateral common carotid arteries (CCAs) were exposed and isolated from the vagus nerves. Microcoils, inner diameter of 0.18 mm (Sawane Spring, Japan), were applied to both CCAs. Sham animals were exposed to the same operative procedures as BCAS mice, except for the application of microcoils. Body temperature was monitored and maintained between 36.5 and 37.5 °C with the aid of a feedback warming pad and a blanket during the operative procedure. Animals were appropriately identified with coded numbers. All of the experiments and data analyses were performed under investigator-blinded conditions. Animals dying during the experimental period and before the planned euthanisation at 16 weeks after surgery were excluded from further analyses. All procedures were pre-approved by the Home Office, London, UK based upon ASPA: The Animals (Scientific Procedures) Act 1986, UK and performed in accordance with the guidelines stipulated by the ethical committee of Newcastle University and adhering to ARRIVE guidelines.

### Environmental enrichment (EE) paradigm and animal groups

Animal were divided into two main groups, sham and BCAS (Fig. 1b). One week after surgery, mice were randomly assigned to six subgroups: Sham-std, sham-operated mice with standard housing; Sham-3 h, sham-operated mice with limited exposure to EE: Sham-full, sham-operated mice with full-time exposure to EE; BCAS-std, BCAS operated mice with standard housing; BCAS-3 h, BCAS operated mice with limited exposure to EE and BCAS-full, BCAS operated mice with full-time exposure to EE. Standard housing (Fig. 1c(A)) denotes a normal housing condition, which incorporated a paper house and shredded tissue. EE cages (Fig. 1c(B)) had extra toys in addition to the standard housing, e.g. running wheels, hanging chains, igloos and a paper tunnel. Limited exposure to EE was performed as described previously [39]. Briefly, for the first 4 weeks, mice were transferred to the EE cages for 3 h every day in the morning from 9 am to 12 pm. From 5th week to 12th week, mice experienced EE cages for 3 h, 3 days a week. Full-time exposure to EE group experienced an EE environment every day for 24 h over the entire 12 weeks. In this study, the experimental animals (survivors) were divided into six groups as follows: Sham-std, sham-operated mice with standard housing, *n =* 11; Sham-3 h, sham-operated mice with intermittent exposure to EE, *n =* 11; Sham-full, sham-operated mice with full-time exposure to EE, *n =* 11; BCAS-std, BCAS operated mice with standard housing, *n =* 13; BCAS-3 h, BCAS operated mice with intermittent exposure to EE, *n =* 10; BCAS-full, BCAS operated mice with full-time exposure to EE, *n =* 12.

### Nesting behaviour

The Nestlets™ (Ancare, USA) test was used to assess the nesting ability of mice [40] before BCAS or sham surgery (as baseline) as well as 16 weeks after surgery before euthanasia. Briefly, one Nestlet, composed of a pressed square of cotton material (2 × 2 in.) was placed at one corner of each cage at 4 pm and left there overnight. First, mice shredded the tightly packed cotton material and then arranged it into a nest. Next morning at 10 am, each cage was assessed in terms of the appearance of the nest (Nestlet score), height of the nest (cm) and the percentage Nestlet used (%). Scoring was defined as follows [40]: 1, Nestlet not noticeably touched (more than 90% intact); 2, Nestlet partially torn (50–90% remaining intact); 3, Nestlet mostly shredded but often no identifiable nest site: less than 50% of the Nestlet remains intact, but less than 90% is within a quarter of the cage floor area, i.e. the cotton is not gathered into a nest but is spread around the cage. The material may sometimes be in a broadly defined nest area, but the critical definition is that 50–90% is shredded; 4, An identifiable but flat nest: >90% of the Nestlet is torn and the material is gathered into a nest within a quarter of the cage floor area, but the nest is flat, with walls higher than the average mouse BW for less than 50% of its circumference; 5, A (near) perfect nest: more than 90% of the Nestlet is torn and the nest is a crater, with walls higher than the average mouse BW for >50% of its circumference.

### Cognitive function assessed by an innovative three-dimensional nine-arm radial maze

Cognitive function (mainly working memory) was assessed in each mouse from 13 weeks post BCAS surgery during a period of 20 days, using an innovative three-dimensional (3D) nine-arm radial maze (3D-RAM) [41]. This was a modified version of the conventional eight-arm radial maze. Behavioural tests were performed in a dimly lit behavioural testing room. The 3D maze (grey PVC, 5 mm thick) consisted of nine arms connected to bridges (slope part) radiating from a central platform. Each arm (35 cm × 11.2 cm) was attached to a bridge (slope part at 40°, 15.2 cm × 11.2 cm). The surface of the bridge was made of metal mesh, which enabled mice to maintain grip. A small transparent wall panel (9 cm × 6 cm) was randomly placed at each entrance of a bridge in order to narrow entry, which avoids a continuous sequential entry from one bridge to the next. In order to enter each arm, mice paced in the centre platform and needed to cross a bridge (elevated ramp) [41]. At the end of each arm, a small pellet (Dustless Precision Pellets® Rodent, Purified, Bio Serv, USA) was placed in order to entice food-restricted mice to enter the arm. Different colours/shapes of pictures were placed vertically at the end of each arm as visual cues, enabling mice to distinguish individual arms. The maze was placed at the centre of a behavioural test room affixed during the entire testing period. All of the sessions for each mouse were streamed and recorded using a camcorder (LEGRIA HF R56, Canon), suspended directly over the maze, via a Wi-Fi network to an iPad (Apple Inc., USA), which was sited outside the behavioural test room.

A day before the first session, each mouse was weighed to ascertain body weight and food deprivation was induced to achieve 10% reduction in their body weight. Mice were weighed immediately prior to each testing session to ensure their BW was maintained at 90% of baseline level. Mice were randomly tested for 20 consecutive days without prior habituation to the maze [42, 43]. Mice placed into a transparent plastic beaker (7 cm diameter, 18.5 cm height) were gently introduced on the central platform at the start of each session. The session was terminated when each mouse completed nine-arm entries or when 10 min had elapsed. After each session, the mouse was allowed to return to the central platform before removing it from the maze in a similarly gentle manner. Between each session, the maze was cleaned with hypochlorous acid-soaked tissue followed by distilled water to remove any trace smells left by urine or faeces. The number of arm entries before first repeat was recorded in each session. The number of arm entries before first repeat in every consecutive 10 sessions and overall 20 sessions were averaged.

### Histopathological analysis

Mice were anaesthetised by intraperitoneal injection of sodium pentobarbital (50 mg/kg). Mice were perfused transcardially at 20 ml/min with 0.01 M phosphate-buffered saline (PBS), pH 7.4. The right or left hemisphere of each brain was randomly selected for histological analysis and subsequently post-fixed in 4% paraformaldehyde (PFA) in 0.01 M PBS (pH 7.4) for 48 h. Brains were cut into five blocks at different coronal levels after fixation: block OB, coronal level of olfactory bulb; block 1, coronal level of bregma +0.5 mm; block 2, coronal level of bregma −1.0 mm; block 3, coronal level of bregma −2.0 mm; block 4, level of cerebellum and brain stem. Each fixed sub-dissected brain block was dehydrated and embedded in paraffin. Five-micrometer-thick sections were cut using a rotary microtome and then mounted on glass slides, stained and analysed.

### Immunohistochemistry/immunofluorescence

#### Immunohistochemistry

Five-micrometer-thick sections obtained from block 3; coronal level of bregma +2.0 mm were used for immunohistochemical analysis of each brain. Immunohistochemistry for glial fibrillary acid protein (GFAP), a marker of astrocytes and ionised calcium-binding adapter molecule-1 (Iba-1), a marker of microglial were performed. Increased GFAP immunoreactivity was used as a marker for astrocytic activation. Antigen retrieval was performed by using 12 min heating in a microwave oven with citrate buffer, pH 6.0 before the sections were then quenched with 3% hydrogen peroxide in phosphate-buffered saline (PBS). Tissues were blocked with normal goat serum, which was derived from the species in which the secondary antibody was generated, for 30 min. After the blocking process, sections were treated with primary antibodies against GFAP (1:2000, DAKO) and Iba-1 (1:200, Wako, Osaka, Japan), 4 °C overnight followed by incubation with an appropriate secondary antibody (biotinylated anti-IgG; 1:200, Vector Laboratories, USA) for 1 h at room temperature. Visualisation for standard colour immunohistochemistry was performed using the Vectastain ABC System (Vector Laboratories) for 30 min at room temperature.

#### GFAP-positive astrocytes and clasmatodendorocytes

Images of GFAP-stained cells within the entire corpus callosum (CC) were captured using a bright field microscope (Leitz DIALUX 20, Leica) with a ×20 objective lens coupled to a lumenera infinity digital camera (Lumenera Corporation, Canada). All GFAP-positive astrocytes and clasmatodendrocytes (damaged astrocytes), characterised by enlarged cell bodies with short processes [44], from each image were counted to assess the total number of astrocytes and clasmatodendrocytes. Using ImageJ software, each CC area was traced and measured (pixels and mm^2^) to calculate percentage of the GFAP-stained area per entire CC area (pixels/pixels, %) as well as numerical density of GFAP-positive astrocytes and clasmatodendrocytes (/mm^2^). Percentage of GFAP-positive clasmatodendrocytes per total number of GFAP-positive astrocytes (%) was also calculated. Furthermore, the numerical density of GFAP-positive clasmatodendrocytes or percentage of GFAP-positive clasmatodendrocytes per total GFAP-positive astrocytes, and CC volume were correlated.

#### Iba-1-positive microglia and microglial activation

Images of Iba-1-stained cells were captured as described previously, using a bright field microscope and a digital camera. All Iba-1-positive microglia from each image were counted to assess the total number of microglia in the entire CC. In addition, all of the Iba-1-positive microglia with the minimal diameter of their cell bodies exceeding 7 or 10 μm (activated microglia) and less than 7 μm (non-activated microglia) were counted to assess the degree of microglial activation. Detailed analysis of microglial morphology, i.e. enlarged cell bodies with thicker and less radially projecting processes was considered to define microglial activation. Using ImageJ software, each CC area was traced and measured (pixels and mm^2^) to calculate percentage of Iba-1-stained area per CC area (pixels/pixels, %) as well as numerical density of Iba-1-positive microglia, Iba-1-positive activated and non-activated microglia (/mm^2^) in the entire CC.

#### Immunofluorescence

Sections obtained from block 3, coronal level of bregma +2.0 mm, were used for immunofluorescent analysis of each brain. Double immunofluorescent staining for GFAP and aquaporin-4 (AQP4) was performed. Five-micrometer-thick brain sections were dewaxed and rehydrated. Antigen retrieval and blocking were performed using the exact same procedure previously described for immunohistochemical staining. After the blocking process, sections were treated with primary antibodies against GFAP (1:2000, DAKO) and AQP4 (1:50, Proteintech), 4 °C overnight followed by incubation with an appropriate secondary antibody (Dylight 650 conjugated anti-IgG, 1:100, Thermo Scientific, for GFAP; Texas Red conjugated anti-IgG, 1:200, Life Technologies, for AQP4) for 1 h at room temperature. Images were captured from the CC using confocal microscopy (TCS SP8, Leica microsystems). Ten consecutive levels were imaged (Z-stacks) through the section, and a merged 2D image was generated. Distribution of AQP4 immunoreactivity in each cell was analysed, and percentage of AQP4 dislocation was calculated in the entire CC.

### Statistical analysis

Data are expressed as mean ± SEM. Using IBM SPSS statistics 22 software, non-paired *t*-test or one-way analysis of variance (ANOVA) followed by post-hoc Tukey’s test were performed for multiple comparison of each group. Pearson’s correlation analysis was performed for assessing correlation between clasmatodendrosis and CC volume. *P* < 0.05 was defined as statistically significant in all analyses.

## Results

### Survival rate of animals in this study

All sham-operated mice (*n =* 33) survived until 16 weeks after surgery. However, survival rate of the BCAS mice was 85.4% (35 of original 41 mice) at 16 weeks after surgery. The survival rates of the BCAS subgroups were as follows: BCAS-std 92.9% (13 of 14), BCAS-3 h 91.0% (10 of 11); BCAS-full 75% (12 of 16). The six causes of deaths in the BCAS group were cerebral haemorrhage (*n =* 1), severe enterocolitis (*n =* 1), kidney anomaly (*n =* 1) and acute renal failure due to dehydration (*n =* 3). The final number of survivors in each group was Sham-std, *n =* 11; Sham-3 h, *n =* 11; Sham-full, *n =* 11; BCAS-std, *n =* 13, BCAS-3 h, *n =* 10; BCAS-full, *n =* 12.

### Effects of EE on astrogliosis and clasmatodendrosis in CC

**Fig. 2:**
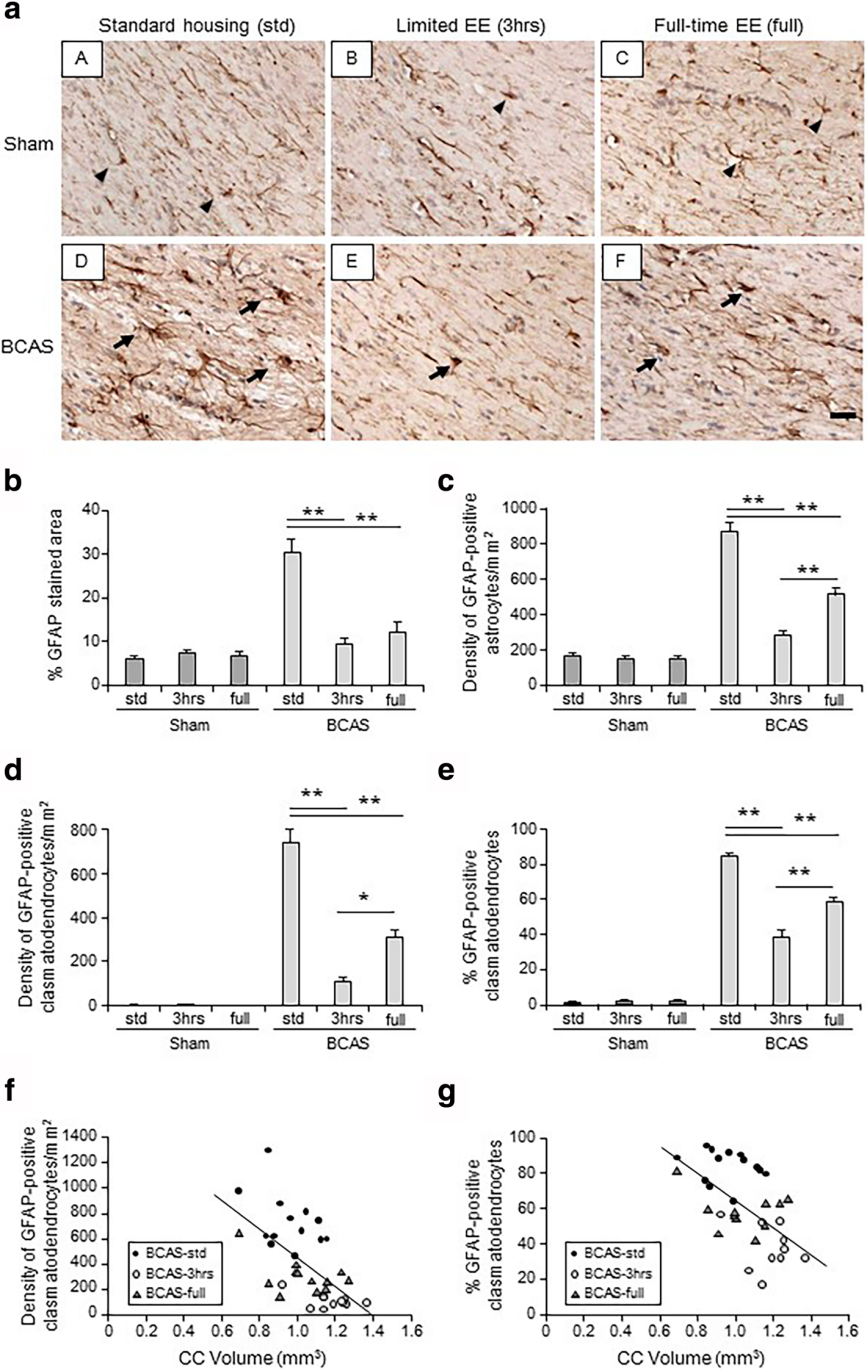
Assessment of astrogliosis and clasmatodendrosis in the corpus callosum (CC). **a**(*A*–*F*) Representative images of GFAP-stained corpus callosum (CC) in each group. **a**(*A*) Sham-std; **a**(*B*) Sham-3 h; **a**(*C*) Sham-full; **a**(*D*) BCAS-std; **a**(*E*) BCAS-3 h; **a**(*F*) BCAS-full. *Scale bar* represents 25 μm. **a**(*A*–*C*) *Arrow heads* indicate normal astrocytes. **a**(*D*–*F*) *Arrows* indicate clasmatodendrocytes. **b**–**c**, Histograms showing GFAP-positively stained astrocytes per area of CC (% GFAP-stained area) (**b**) as well as numerical density of GFAP-positive astrocytes in the entire CC (**c**). BCAS-3 h showed decreased GFAP-stained area and decreased numerical density of GFAP-positive astrocytes in the entire CC compared with BCAS-std (**P < 0.01). BCAS-full showed decreased GFAP-stained area in the entire CC compared with BCAS-std (**P < 0.01) (**b**, **c**). BCAS-3 h also showed decreased numerical density of GFAP-positive astrocytes in the entire CC compared with BCAS-full (**P < 0.01) (**c**). **d**, **e** Histograms showing numerical density of GFAP-positive clasmatodendrocytes (damaged astrocytes) in the entire CC (**d**) and percentage of clasmatodendrocytes per total number of GFAP-positive astrocytes in the entire CC (**e**). BCAS-3 h and BCAS-full showed reduced numerical density of GFAP-positive clasmatodendrocytes in the entire CC and % of clasmatodendrocytes per all GFAP-positive astrocytes compared with BCAS-std (**P < 0.01) (**d** and **e**). BCAS-3 h also showed reduced numerical density of GFAP-positive clasmatodendrocytes in the entire CC compared with BCAS-full (*P < 0.05) (**d**) and % of clasmatodendrocytes per all GFAP-positive astrocytes compared with BCAS-std (**P < 0.01) (**e**). **f**, **g** Histograms showing negative correlation between numerical density of clasmatodendrocytes and CC volume (**f**) as well as correlation between % of clasmatodendrocytes and CC volume (**g**) in the CC of BCAS cohort. Pearson’s correlation analysis revealed that degree of clasmatodendrosis in the CC of BCAS cohort exhibited strong negative correlation with CC atrophy (*r =* 0.615, *P* < 0.01, between numerical density of clasmatodendrocytes and CC volume, (**f**); *r =* 0.583, *P* < 0.01, between % of clasmatodendrocytes in the CC and CC volume, (**g**)). Sham-std, *n =* 11; Sham-3 h, *n =* 11; Sham-full, *n =* 11; BCAS-std, *n =* 13, BCAS-3 h, *n =* 10; BCAS-full, *n =* 12

BCAS induced astrogliosis, characterised by increased percentage of GFAP-stained area as well as increased numerical density of GFAP-positive astrocytes in the entire CC (Fig. 2a–c). This was evident as both hypertrophic and hyperplastic responses. Limited exposure to EE (BCAS-3 h) reduced % GFAP-stained area compared with BCAS-std (*P* < 0.01) (Fig. 2b) and the numerical density of GFAP-positive astrocytes in the entire CC compared with BCAS-std and BCAS-full subgroups (both *P* < 0.01) (Fig. 2c). BCAS-full also showed reduced % GFAP-stained area and numerical density of GFAP-positive astrocytes in the entire CC compared with BCAS-std (both *P* < 0.01) (Fig. 2b, c). There were no differences between BCAS-3 h and all sham subgroups in % GFAP immunoreactivity per whole CC area (Fig. 2b, c). BCAS-full subgroup showed no difference between all Sham subgroups in % GFAP-stained area per whole CC area (Fig. 2b). BCAS also induced clasmatodendrosis: significantly increased damaged astrocytes in the CC (Fig. 2d, e). Limited exposure to EE (BCAS-3 h) subgroup exhibited reduced density of GFAP-positive clasmatodendrocytes in the entire CC and % of clasmatodendrocytes per all GFAP-positive astrocytes compared with BCAS-std (*P* < 0.01) (Fig. 2d, e). BCAS-3 h subgroup also showed reduced density of GFAP-positive clasmatodendrocytes compared with BCAS-full (*P* < 0.05) (Fig. 2d) and % of clasmatodendrocytes per all GFAP-positive astrocytes compared with BCAS-std (*P* < 0.01) (Fig. 2e). BCAS-full showed reduced density of GFAP-positive clasmatodendrocytes as well as reduced % of clasmatodendrocytes per all GFAP-positive astrocytes compared with BCAS-std (Fig. 2d, e). There were no differences between BCAS-3 h and all sham subgroups in the density of GFAP-positive clasmatodendrocytes (Fig. 2d).

### Degree of clasmatodendrosis correlated with CC atrophy

Increased clasmatodendrosis in the atrophied CC was most evident in BCAS-std followed by BCAS-full across BCAS subgroups. Limited exposure to EE (BCAS-3 h) tended to show less clasmatodendrosis in the relatively preserved larger CC (Fig. 2f, g). The density of clasmatodendrocytes was negatively correlated with CC volume (*r =* 0.615, *P* < 0.01) (Fig. 2f). Percentage of clasmatodendrocytes per all GFAP-positive astrocytes was also negatively correlated with CC volume in the BCAS cohort (*r =* 0.583, *P* < 0.01) (Fig. 2g).

### Abnormal distribution of aquaporin-4 (AQP4) within GFAP-positive astrocytes

**Fig. 3:**
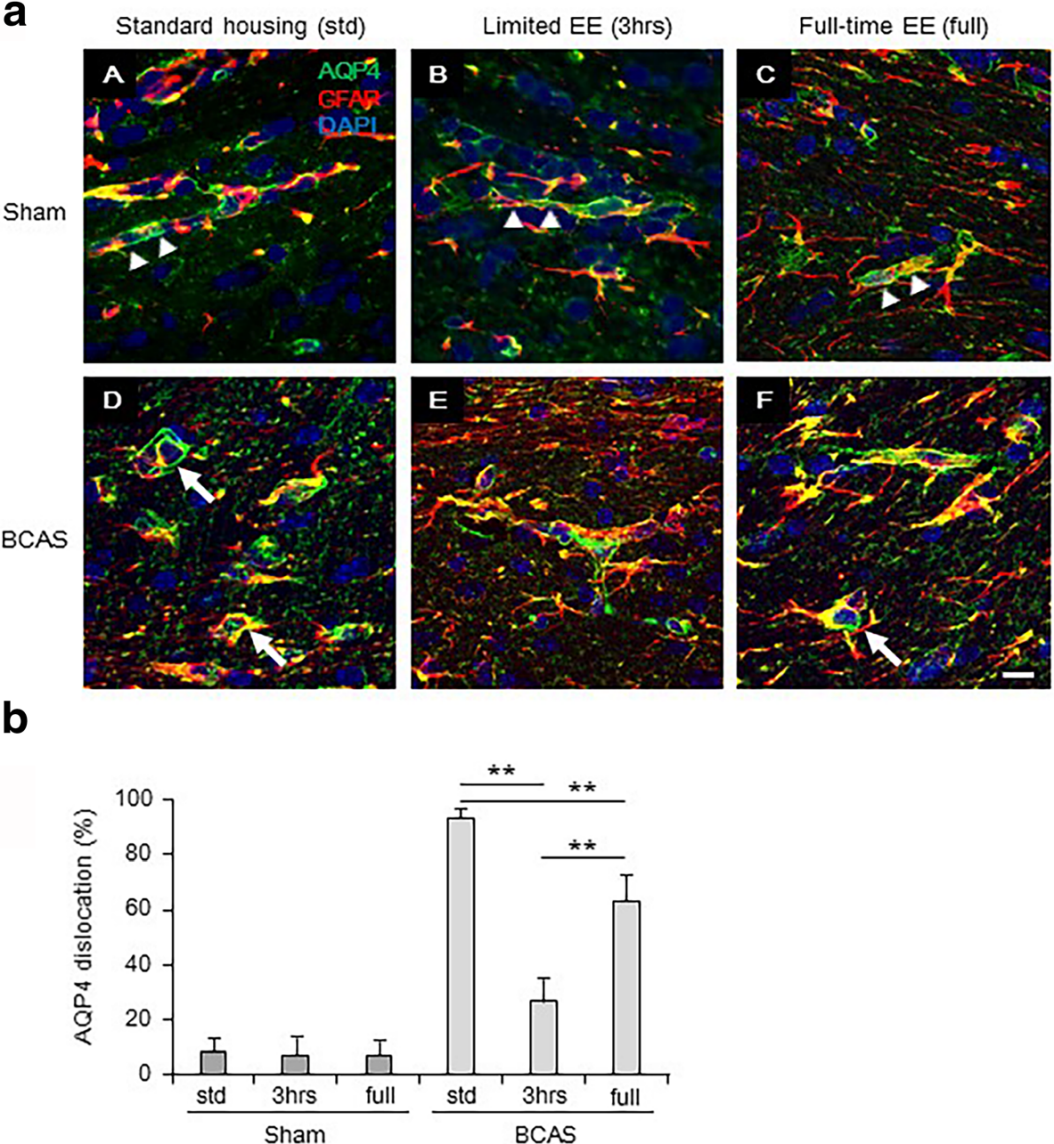
GFAP and AQP4 distribution in the corpus callosum (CC). **a**(*A*–*F*) Representative images of double immunofluorescent staining for GFAP and AQP4 in the corpus callosum (CC). **a**(*A*) Sham-std; **a**(*B*) Sham-3 h; **a**(*C*) Sham-full; **a**(*D*) BCAS-std; **a**(*E*) BCAS-3 h; **a**(*F*) BCAS-full. *Scale bar* represents 10 μm. **a**(*A*-*C*) In Sham subgroups, AQP4 was normally distributed within the astrocytic end-feet around the vessels (*arrow heads*). **a**(*D*) and **a**(*F*) In BCAS subgroups, AQP4 was abnormally aggregated at the periphery of GFAP-positive astrocytes/clasmatodendrocytes, especially in BCAS-std and BCAS-full subgroups (*arrows*). **a**(*E*) In limited exposure to EE (BCAS-3 h), less abnormal distribution of AQP4 was seen in the CC. **b** Histogram showing % AQP4 dislocation in each group. EE, especially by limited exposure to EE (BCAS-3 h) attenuated AQP4 dislocation in the CC (**P < 0.01). Sham-std, *n =* 11; Sham-3 h, *n =* 11; Sham-full, *n =* 11; BCAS-std, *n =* 13, BCAS-3 h, *n =* 10; BCAS-full, *n =* 12

In sham subgroups, aquaporin-4 (AQP4) was normally distributed within GFAP-positive astrocytic end-feet around the vessels (Fig. 3a(A–C)). Abnormal distribution of AQP4, characterised by loss or retraction of the astrocytic end-feet around the vessels and abnormal aggregation of AQP4 at the periphery of GFAP-positive astrocytes/clasmatodendrocytes, was most evident in the BCAS-std animals followed by BCAS-full (Fig. 3a(D, F). Limited exposure to EE (BCAS-3 h) effectively reduced the abnormal distribution of AQP4 in the CC (Fig. 3a(E), b).

### Microglial proliferation and activation induced by BCAS

**Fig. 4:**
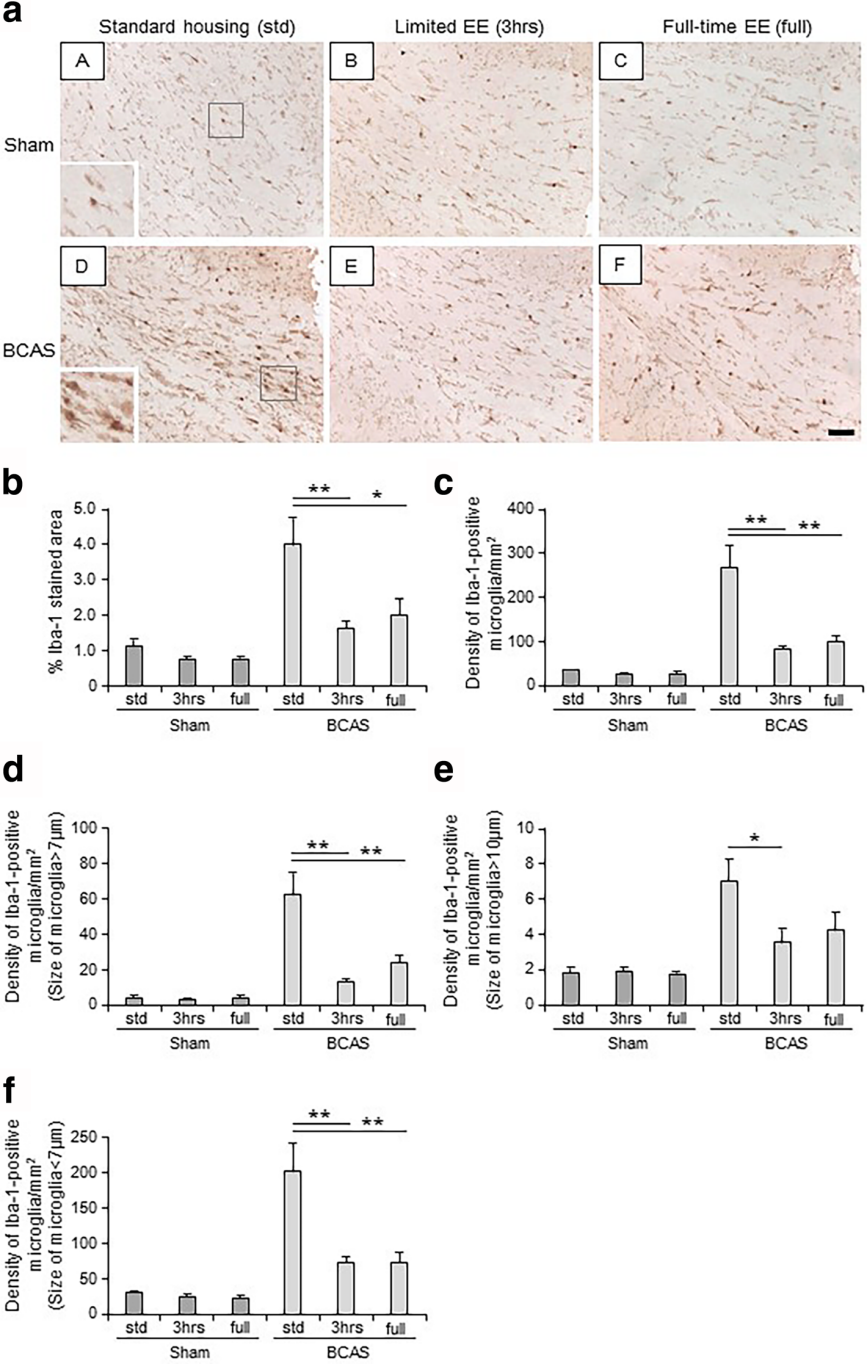
Assessment of microglial proliferation and activation in the corpus callosum (CC). **a** (*A*–*F*) Representative images of Iba-1 stained corpus callosum (CC) in each group. **a**(*A*) Sham-std; **a**(*B*) Sham-3 h; **a**(*C*) Sham-full; **a**(*D*) BCAS-std; **a**(*E*) BCAS-3 h; **a**(*F*) BCAS-full. Scale bar represents 50 μm. **a**(*A*) Inset showing a normal microglial cell. **a**(*D*) Inset showing activated microglial cells. **b**, **c** Histograms showing degree of microglial proliferation in each subgroup. EE, especially by limited exposure to EE (BCAS-3 h) suppressed microglial proliferation in the CC induced by BCAS (**P < 0.01). **d**–**f** Histograms showing degree of microglial activation in each subgroup. Limited exposure to EE suppressed degree of microglial activation in both moderate (microglial cell body >7 μm) (**P < 0.01) (**d**) and highly (microglial cell body >10 μm) activated (*P < 0.05) (**e**) levels, as well as suppressed the number of non-activated microglial cells (**P < 0.01) (**f**). However, BCAS-full did not show suppressed highly activated microglia in the CC (**e**). Sham-std, *n =* 11; Sham-3 h, *n =* 11; Sham-full, *n =* 11; BCAS-std, *n =* 13, BCAS-3 h, *n =* 10; BCAS-full, *n =* 12

Microglial proliferation and activation in the CC were evident in BCAS-std subgroup (Fig. 4a–f). Limited exposure to EE (BCAS-3 h) suppressed microglial proliferation, characterised by an increased % Iba-1-stained area per entire CC area and increased density of Iba-1-positive microglia per entire CC area (both *P* < 0.01) (Fig. 4b, c). BCAS-3 h also suppressed degree of microglial activation in both moderately (microglial cell body >7 μm) (*P* < 0.01) (Fig. 4d) and highly (microglial cell body >10 μm) activated (*P* < 0.05) (Fig. 4e) levels, as well as suppressing the number of non-activated microglial cells (*P* < 0.01) (Fig. 4f). BCAS-full also attenuated microglial proliferation and activation (Fig. 4b–f); however, this was more effective in the BCAS-3 h animals than the BCAS-full animals. BCAS-full did not suppress highly activated microglia in the CC compared with BCAS-std (Fig. 4e). There were no differences evident between BCAS-3 h or BCAS-full and all sham subgroups.

### Impaired nesting ability induced by BCAS was reversed by EE

**Fig. 5:**
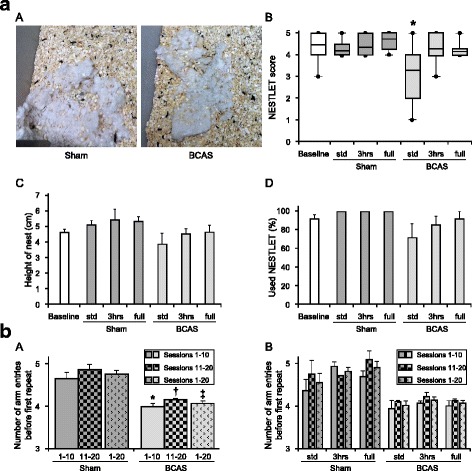
Nesting ability and cognitive function assessed in the nine-arm radial maze [72, 73]. **a**(*A*) Representative images of nest created from the Nestlet in Sham and BCAS group. **a**(*B*) Boxplot showing Nestlet score at baseline level and post operation in each group. **a**(*C*, *D*) Histograms showing height of the nest (*C*) and % used Nestlet (*D*) at baseline level and post operation in each group. BCAS-std subgroup had impaired nesting ability compared to baseline level (*P < 0.05). EE reversed impaired nesting ability induced by BCAS, especially by limited EE (BCAS-3 h) (**a**(*B*)). **b**(*A*, *B*), Histograms showing number of arm entries before first repeat in all sham and all BCAS (*A*) as well as number of arm entries before first repeat of each subgroup (*B*) assessed in the nine-arm radial maze. Every consecutive 10 sessions and overall 20 sessions were averaged. **b**(*A*) All sham completed more arm entries before first repeat, compared with all BCAS in sessions 1–10, sessions 11–20 and sessions 1–20 (*P < 0.01 vs sham; †*P* < 0.01 vs sham; ‡*P* < 0.01 vs sham). **b**(*B*) Between BCAS subgroup, BCAS-3 h completed more arm entries compared with BCAS-std and BCAS-full in sessions 1–10, sessions 11–20 and sessions 1–20. Sham-std, *n =* 11; Sham-3 h, *n =* 11; Sham-full, *n =* 11; BCAS-std, *n =* 13, BCAS-3 h, *n =* 10; BCAS-full, *n =* 12

Sham subgroups had almost the same level of nesting ability (Nestlet score) compared with preoperative baseline level at 16 weeks after surgery (Fig. 5a(A–D)). However, BCAS-std subgroup exhibited lower Nestlet scores (impaired nesting ability) compared to baseline level (*P < 0.05) (Fig. 5a(A, B)). EE reversed impaired nesting ability, and limited exposure to EE (BCAS-3 h) tended to improve nesting ability compared with BCAS-std and BCAS-full subgroups (Fig. 5b). BCAS plus EE subgroups made higher nests, used more Nestlet to make nests compared with BCAS-std (Fig. 5a(C, D)).

### Impaired working memory induced by BCAS was partially reversed by EE

Working memory was significantly impaired in BCAS compared to sham animals. The number of arm entries before first repeat was lower in BCAS mice compared to the sham in sessions 1–10, 11–20 and 1–20 (all *P* < 0.01) (Fig. 5b(A)). All sham subgroups also completed more arm entries before first repeat compared with all BCAS subgroups. Between BCAS subgroups, the BCAS-3 h mice subgroup completed more arm entries before first repeat, in sessions 1–10, 11–20 and 1–20 (Fig. 5b(B)).

## Discussion

We report on the favourable effects of EE on WM glial damage in the BCAS mouse model. We found that chronic cerebral hypoperfusion induced by bilateral common carotid artery stenosis (BCAS) caused (1) astrogliosis, clasmatodendrosis (increased number of clasmatodendrocytes); (2) microglial proliferation/activation in the corpus callosum (CC); and (3) impaired nesting ability and cognitive function. This study provides strong evidence to support the notion that limited EE could suppress brain hypoperfusion-induced glial damage in WM and neuroinflammation in VCI and possibly VaD patients.

Our observations are consistent with recent studies on the pathophysiology of the WM in post-stroke dementia (PSD). We found highly increased degeneration of astrocytes in PSD subjects who had greater WM hyperintensity volumes [45]. Clasmatodendrosis, damaged astrocytes with enlarged cell bodies and loss of processes, was particularly prominent in the deep WM of the frontal lobe [44]. Similarly, increased degree of clasmatodendrosis was evident in the CC of the BCAS group. Astrocytes are sensitive to various stimuli, e.g. ischaemia, and transform themselves to reactive cells upon stimulation. It is known that protoplasmic astrocytes lose their integrity faster than fibrous astrocytes [27, 46]. In the human white matter (WM), most of the astrocytes represent the fibrous form [47]. In our study, the astrogliosis appears to accord with the fibrous type (Fig. 2a), suggesting that fibrous type of astrocytes acquires characteristics of GFAP-positive reactive astrocytes or clasmatodendrocytes after BCAS. Furthermore, we found that the numerical density of clasmatodendrocytes in the CC of the BCAS cohort, and percentage of clasmatodendrocytes per all GFAP-positive astrocytes were negatively correlated with CC volume. Thus, clasmatodendrosis was evident in atrophied and damaged CC induced by cerebral hypoperfusion. This suggests that clasmatodendrosis is caused via vascular changes, e.g. pathological alterations in small arteries and blood-brain-barrier (BBB) disruption [48, 49] following BCAS-related ischaemic insult, which is compatible with the findings in human cerebrovascular disease and Alzheimer’s disease [50]. In reactive astrocytes, p38 mitogen-activated protein kinases (p38 MAPK) is activated, produce reactive oxygen species (ROS) [51] and induce cellular inflammation [52], which contributes to and reinforce an inflammatory cascade. Targeting of astrocytes with pharmacological agents that may return reactive astrocytes to a quiescent phenotype [26] could represent an important therapeutic treatment for CC atrophy.

Water homeostasis in brain is regulated by the water channel family, the aquaporins [53]. In physiological conditions, aquaporin-4 (AQP4) is present on astrocytic end-feet around the vessels in the brain [54] and contributes to water homeostasis and central plasma osmolarity regulation. AQP4 also play a role in pathophysiologic conditions, e.g. reduced edema formation after cerebral ischemia [53, 55]. We previously demonstrated AQP4 immunoreactivity is dislocated in PSD as well as in a non-human primate model of cerebral hypoperfusion [44]. We found similar AQP4 disruption in the CC, compatible with our recent report [56]. Taken together, AQP4 dislocation and clasmatodendrosis caused by BCAS may enhance WM pathology (including CC atrophy) due to the disturbance of water homeostasis [44]. Notably, limited exposure to EE, rather than full-time exposure to EE, significantly attenuated clasmatodendrosis and AQP4 disruption in the mouse model of long-term chronic cerebral hypoperfusion. This suggests limited exposure to EE relatively preserves blood-brain-barrier (BBB) integrity via the maintenance of glio-vascular integrity after BCAS. Long-term BCAS can be considered a relevant model as it accurately mimics glio-vascular pathology seen in VCI/VaD.

BCAS also caused a chronic neuroinflammatory response in the WM (CC), indicated by the upregulation of microglial density in the affected WM region [15, 57]. EE cases of BCAS had considerably reduced microglial activation/proliferation in comparison to non-enriched BCAS-std, which is also a novel finding in the BCAS mouse model. Limited exposure to EE (BCAS-3 h) showed some effects compared to the sham subgroups, which indicated that regular limited exposure to EE showed promise to improve BCAS-induced inflammation to a similar level shown in sham groups.

Upon activation, microglia undergo fundamental morphological changes in response to different environmental stimuli. Microglial activation results in hypertrophy, with thicker and less radially projecting processes [58] and expresses mitochondrial translocator protein (TSPO) [59]. The response of microglia might be dependent on different degrees of activation and may be detrimental [60]. Tumour necrosis factor-alpha (TNF-α) produced by activated microglia plays a critical role in the induction of neuronal death in Alzheimer’s disease [61]. In addition, increased proliferation of microglia was evident in human AD cases with increased colony-stimulating factor 1 receptor (CSF1R) expression, and inhibition of CSF1R attenuated microglial proliferation in a mice model of AD [62]. Previous studies have suggested that chronic over-activation of microglia results in the overproduction of cytotoxic factors such as proteases (i.e. matrix metalloproteinase-2 (MMP-2)), reactive oxygen species and pro-inflammatory cytokines [63–65] that promote neurotoxicity and neuronal damage. Chronic cerebral hypoperfusion induces MMP-2 in microglia [66]. MMP-2 plays a critical role in the BBB disruption, glial activation, and WM lesions after chronic cerebral hypoperfusion [67]. In our study, BCAS-std and BCAS-full demonstrated a greater rate of microglial over-activation, which corresponds to more CC atrophy. Thus, WM damage observed in BCAS mice may occur due to microglial activation-induced neuronal/axonal damage. Identifying molecular targets [68] of microglial activation and microglial suppressant agents such as minocycline [69] and ethyl pyruvate [70] could attenuate microglial activation and protect against brain ischaemia. With this in mind, we surmise that continuous full-time environmental enrichment and no enrichment have likely detrimental effects on WM pathology due to excessive stimulation of microglial over-activation. It is plausible that the exacerbated WM injury during continuous full-time enrichment and no enrichment is partially caused by the neurotoxic effects of microglial over-activation induced by chronic neuroinflammation [71].

It has been reported that nesting is vital for heat conservation, reproduction and shelter and shown to relate to functional status due to brain lesions, pharmacological agents and genetic mutations [40]. We found that BCAS impaired nesting ability, and it was attenuated by EE. BCAS plus limited exposure to EE mice tended to make better nests compared to other BCAS subgroups. These results suggest that EE after chronic cerebral hypoperfusion could protect changes in different domains of functional status or attenuate pathological changes, e.g. subtle sensory-motor, pure-motor deficit and general health conditions involved in nesting ability. We also utilised an innovative 3D nine-arm radial arm maze for the assessment of cognitive function, which is a modified version of the conventional radial arm maze and designed for assessing working memory [42]. There were working memory deficits in the animals with BCAS and improvement in those exposed to EE, particularly by limited exposure to EE [72, 73]. This is consistent with the heterogeneity observed in VaD and normally ageing individuals.

We also suggest the higher mortality in the BCAS-full subgroup is explained by dehydration and acute renal failure possibly due to increased overriding physical activity promoted by the full-time exposure to EE. Although EE exposes the animals to a number of different features including physical exercise and social interaction, we noted the average number of wheel rotations in BCAS-full subgroup was fivefold greater compared with BCAS-3 h, Sham-3 h and Sham-full subgroups (data not shown), probably due to disruption of fronto-subcortical circuit induced by BCAS, which might be a similar symptom of “disinhibition” seen in patients with VaD and frontal lobe dysfunction. Furthermore, as limited exposure to EE group experienced different types of housing condition (change in housing condition) in each time when exposed to EE, limited EE could have initiated and activated the fronto-subcortical circuits more often when the animals experienced EE housing every time. In contrast, full-time exposure to EE group experienced same EE condition for 24 h a day in the entire 12 weeks of experimental period without changes in housing condition. Therefore, full-time EE might not have initiated and activated the fronto-subcortical circuit effectively compared with limited EE. Repeated activation of fronto-subcortical circuits induced by limited EE could enhance cognitive function and prevent cognitive decline after BCAS. These observations may explain why limited exposure to EE was more beneficial than full-time exposure to EE.

One of the few limitations of our study is that we assessed glial activation/proliferation only at a single time point, 16 weeks after surgery. Histological data obtained from several time points, while cumbersome would be useful to understand the temporal profile of glial cell responses as well as effects of EE against astrogliosis and microglial activation/proliferation in BCAS. Another limitation is that we only set limited exposure to EE regime at 3 h a day. Testing other durations of limited EE, such as shorter than 3 h will elucidate the optimal effects of limited exposure to EE against gliosis and neuroinflammation over a period of time, as little is known of EE for the most effective way to modify these pathologies. Finally, greater analysis of all the WM regions of the cerebrum including the anterior commissure may have informed on how widespread the described glial responses occur due to BCAS or cerebral hypoperfusion, which leads to VCI or VaD.

## Conclusions

In summary, long-term chronic cerebral hypoperfusion induced by BCAS produced similar glial damage, glial activation and proliferation as evident in VaD. Our study also demonstrated that limited exposure to EE effectively suppressed adverse glial activation/proliferation, preserved neurovascular/oligovascular units and likely BBB integrity after chronic cerebral hypoperfusion-induced vascular insults. By modifying detrimental glial inflammatory responses implementing limited EE and even (poly) pharmacological approaches might be a safe and effective interventional strategy for cerebrovascular disorders which lead to dementia.
